# Correlation between serum complement component 4 levels and tubular atrophy in patients with lupus nephritis

**DOI:** 10.1080/0886022X.2025.2477833

**Published:** 2025-03-17

**Authors:** Zheyi Chang, Haofei Hu, Yuan Cheng, Ricong Xu, Qijun Wan

**Affiliations:** Department of Nephrology, the First Affiliated Hospital of Shenzhen University (The Second People’s Hospital of Shenzhen), Shenzhen, China

**Keywords:** Chronic kidney disease, complement component 4, lupus nephritis, systemic lupus erythematosus, tubular atrophy

## Abstract

**Objective:**

The aim of this study is to examine the relationship between serum complement component 4 (C4) levels and tubular atrophy in patients with lupus nephritis (LN).

**Methods:**

Patients diagnosed with LN through renal biopsy were retrospectively analyzed and categorized into two groups: the tubular atrophy group (TA group) and the non-tubular atrophy group (non-TA group). Demographic data, clinical characteristics, and pathological findings were compared between the groups. Logistic regression and spline smoothing plot analysis, utilizing the generalized additive mixed model, were employed to investigate the relationship between serum C4 levels and the occurrence of tubular atrophy.

**Results:**

A total of 129 patients were included in the study, with 57 (44.2%) identified as having tubular atrophy (TA group), while the remaining 72 patients did not present with tubular atrophy (non-TA group). Patients in the TA group exhibited higher serum C4 levels (*p* = 0.008) and lower eGFR (*p* = 0.001) compared to those in the non-TA group. Pathological findings revealed that the TA group had a higher incidence of mesangial hypercellularity (*p* = 0.041), endocapillary hypercellularity (85.96% vs. 68.06%, *p* = 0.018), interstitial inflammation (*p* < 0.018), karyorrhexis (78.95% vs. 59.72%, *p* = 0.02), and arteriole hypertrophy (66.67% vs. 38.89%, *p* = 0.02). Multivariate logistic regression analysis revealed that elevated serum C4 levels were associated with tubular atrophy in patients with LN (OR = 1.069, 95% CI 1.001 − 1.141, *p* = 0.048). Furthermore, spline smoothing analysis using the generalized additive mixed model indicated a linear relationship between serum C4 levels and the occurrence of tubular atrophy events. This linear relationship remained consistent across various stratifications, including age, hypertension, leukocyte count, hemoglobin levels, and estimated glomerular filtration rate (eGFR).

**Conclusion:**

Serum C4 levels demonstrate a significant correlation with the presence of tubular atrophy in patients diagnosed with LN.

## Introduction

Systemic lupus erythematosus (SLE) is a multisystem autoimmune disorder, with lupus nephritis (LN) being among its most severe complications. LN is a significant contributor to the development of end-stage renal disease (ESRD), imposing a substantial global health burden.

Previous studies have identified tubular atrophy as an independent risk factor for poor renal outcomes in patients with LN. Tubular atrophy represents a pathological process characterized by the loss of tubular epithelial cells and subsequent structural changes in renal tubules. This condition typically results from chronic injury to the tubular epithelium, leading to reduced tubular function and decreased renal capacity for filtration, reabsorption, and secretion. The presence of significant tubular atrophy often indicates chronic kidney damage and is associated with progressive decline in renal function [1,2]. In the context of LN, tubular atrophy may result from various mechanisms including immune complex deposition, inflammatory mediators, and complement activation. The activation of the complement system is recognized as a critical factor in the renal injury associated with SLE; however, the precise role of the complement system in the pathogenesis of SLE remains a topic of debate. On one hand, genetic deficiency of complement component C4 has been suggested to increase susceptibility to the development of SLE [3]. On the other hand, the activation of C4 by immune complexes (ICs) is known to exacerbate tissue injury in patients with SLE [4].

In IgA nephropathy, now increasingly recognized by some physicians as an autoimmune kidney disease, previous studies have demonstrated that low serum complement 4 (C4) levels are associated with milder tubular atrophy and interstitial fibrosis [5–7]. Besides, recent studies show that there was a strong correlation between the plasma C4d level and the plasma C4d/C4 ratio as well as C4d deposition in renal biopsy samples from LN patients, who presented with higher Chronic Indices (CI) scores [8,9]. Considering the short half-live and the difficulties on measurement of the C4d, we tried to find out whether there was correlation between the serum C4 and tubular atrophy- recognized as a part of CI scores. However, in patients with LN, the relationship between serum C4 levels and tubular atrophy—recognized as an independent risk factor for chronic kidney disease (CKD)—has not been thoroughly investigated. To address this gap, we conducted a cross-sectional study among of Chinese patients with LN to evaluate the association between serum C4 levels and both their clinical and histopathologic characteristics.

## Methods

### Study participants

This cross-sectional study focused on analyzing the relationship between C4 levels and tubular atrophy at the time of renal biopsy. It was a single-center, cross-sectional analysis involving patients aged 14 years and older with biopsy-confirmed LN who received treatment at the First Affiliated Hospital of Shenzhen University between January 2010 and February 2019 were included. Due to the retrospective design of the study and the maintenance of patient anonymity through the exclusion of identifiable information, the requirement of informed consent was waived. The study received approval from the Medical Ethics Committee of Shenzhen Second People’s Hospital and was conducted in accordance with the ethical standards outlined in the World Medical Association Declaration of Helsinki.

### Collection of clinical and histologic variables

Demographic data, including age and gender, as well as clinical parameters such as blood pressure (BP), body weight, and height, were recorded on the day of the renal biopsy. Laboratory data, including estimated glomerular filtration rate (eGFR) calculated using the Modification of Diet in Renal Disease (MDRD) equation, 24-h urine protein levels, hemoglobin, platelet count, serum albumin, low-density lipoprotein (LDL), serum C3, and serum C4, were collected at the time of the renal biopsy. All renal biopsy specimens were independently reviewed by two experienced renal pathologists from Guangzhou KingMed Center using light microscopy, immunofluorescence, and electron microscopy (EM). To maintain objectivity, two pathologists used a uniform scoring system, with any discrepancies resolved through consensus discussion or, if necessary, consultation with a third independent pathologist. To ensure comprehensive and consistent evaluation, each biopsy specimen was required to include at least 10–15 glomeruli, with a representative sampling of different renal tissue areas. The pathologists followed standardized protocols to minimize inter-observer variability and ensure high-quality tissue assessment.

The clinical laboratory findings were classified according to the International Society of Nephrology/Renal Pathology Society (ISN/RPS) 2003 Classification. Tubular atrophy was defined as the presence of more than 5% of tubules exhibiting loss of cytoplasmic organelles, a reduced tubular diameter, and thickening of the tubular basement membrane (TBM).

### Statistical analysis

Statistical analysis was conducted using a rigorous, multi-step approach. Continuous variables were first assessed for normality using the Shapiro–Wilk test (*p* > 0.05 considered normally distributed). Normally distributed data were presented as mean ± standard deviation, while non-normally distributed data were reported as median (interquartile range). Statistical test selection was based on data distribution, sample size, and underlying research hypotheses, ensuring the most appropriate and robust analytical method was employed for each specific analysis. Categorical variables were expressed as frequencies or percentages. Statistical differences between groups were assessed using One-Way ANOVA for normally distributed data, Kruskal–Wallis H test for skewed data, and chi-square tests for categorical variables.

Univariate linear regression models were employed to evaluate the association between serum C4 levels and tubular atrophy in patients with LN. Both unadjusted and multivariate adjusted models were reported. Adjustments were made based on whether adding a covariate to the model altered the odds ratio by at least 10% [10]. Additionally, a generalized additive model (GAM) was used to explore potential non-linear relationships. If non-linearity was detected, a two-piecewise linear regression model was applied to assess the threshold effect of C4 on tubular atrophy using spline smoothing analysis.

Subgroup analyses were conducted with stratified linear regression models, and modifications and interactions within subgroups were examined using the likelihood ratio test. All analyses were performed using EmpowerStats (http://www.empowerstats.com, X&Y Solutions, Inc., Boston, MA, USA). A *p-*value of less than 0.05 (two-sided) was considered statistically significant.

## Results

### Participant demographics

A total of 129 patients with LN were classified into two groups based on renal biopsy diagnosis: the tubular atrophy group (57 patients, 44.2%) and the non-tubular atrophy group (72 patients, 55.8%). Approximately 91% of the patients were female. The patients in the tubular atrophy group were older compared to those in the non-tubular atrophy group (mean age 36.98 ± 12.29 years vs. 30.47 ± 9.67 years, *p* = 0.001). Additionally, the tubular atrophy group exhibited a higher systolic blood pressure (133.70 ± 21.99 mmHg) compared to the non-tubular atrophy group (123.85 ± 19.59 mmHg), though this difference did not reach statistical significance (*p* = 0.08).

### Baseline clinical and histopathological features

The clinical and histopathological features of the patients at the time of renal biopsy are summarized in Table 1. The tubular atrophy group exhibited significantly lower eGFR (*p* = 0.001) and higher serum C4 levels (*p* = 0.008) compared to the nontubular atrophy group. Additionally, the non-tubular atrophy group had higher hemoglobin levels (*p* = 0.033). No significant differences were observed in 24-h urine protein levels, serum albumin, serum uric acid, LDL, serum C3 levels, anti-dsDNA antibody, or anti-Sm antibody between the two groups.

**Table 1.** Characteristics of LN patients with tubular atrophy compared with those without tubular atrophy.

Clinical characteristics	Non-TA group *N* = 72	TA group *N* = 57	*p* Value
Sex, female (n [%])	66 (91.67%)	51 (89.47%)	0.670
Age (years, mean ± SD)	30.47 ± 9.67	36.98 ± 12.29	0.001
Systolic blood pressure (mmHg, mean ± SD)	123.85 ± 19.59	133.70 ± 21.99	0.008
Diastolic blood pressure (mmHg, mean ± SD)	79.76 ± 11.27	83.96 ± 14.29	0.064
24h urinary protein [g, median (interquartile range)]	2.14 (1.06-3.93)	2.62 (1.25-5.16)	0.072
Hemoglobin	106.02 ± 20.22	97.63 ± 24.02	0.033
Leukocyte (×10^9^ /L, median (interquartile range))	4.51 (3.48-6.31)	5.60 (4.01-8.10)	0.041
Platelet (×10^9^ /L, mean ± SD)	179.73 ± 85.00	186.37 ± 77.84	0.649
Serum albumin (g/L, mean ± SD)	26.18 ± 7.81	26.96 ± 8.23	0.582
eGFR (ml/min/1.73 m^2^, mean ± SD)	100.60 ± 37.26	76.49 ± 45.58	0.001
Serum uric acid (μmol/L, mean ± SD)	404.02 ± 127.00	436.41 ± 151.51	0.189
LDL (mmol/L, mean ± SD)	3.51 ± 1.57	3.26 ± 1.54	0.389
Serum C3 (g/L, mean ± SD)	0.55 ± 0.28	0.61 ± 0.26	0.214
Serum C4 (g/L, median (interquartile range))	0.04 (0.01-0.11)	0.07 (0.04-0.12)	0.008
SLEDAI score (mean ± SD)	14.67 ± 6.40	15.23 ± 5.85	0.609
CRP (mg/L mean ± SD))	8.4 ± 16	4.4 ± 6.4	0.101
SSA	42 (58.33%)	39 (68.42%)	0.239
SSB	15 (20.83%)	13 (22.81%)	0.787
Anti-dsDNA (n [%])	56 (77.78%)	39 (68.42%)	0.231
Anti-SM antibody (n [%])	23 (31.94%)	12 (21.05%)	0.167
Histological characteristics			
Lupus nephritis class, (n [%])			0.362
I	1 (1.39%)	0 (0.00%)	
II	10 (13.89%)	5 (8.77%)	
III	10 (13.89%)	8 (14.04%)	
IV	27 (37.50%)	24 (42.11%)	
V	12 (16.67%)	4 (7.02%)	
III+V	1 (1.39%)	3 (5.26%)	
IV+V	11 (15.28%)	13 (22.81%)	
Global glomerulosclerosis (%, mean ± SD)	1.67 ± 4.33	13.70 ± 19.13	<0.001
Segmental glomerulosclerosis (n [%])	1 (1.39%)	10 (19.3%)	0.002
Endocapillary hypercellularity (n [%])	49 (68.06%)	49 (85.96%)	0.018
Mesangial hypercellularity (n [%])			0.041
None (<5%)	1 (1.39%)	1 (1.75%)	
Mild (5%< =∼<25%)	35 (48.61%)	14 (24.56%)	
Moderate (25%< =∼<50%)	24 (33.33%)	25 (43.86%)	
Severe (>50%)	12 (16.67%)	17 (29.82%)	
Karyorrhexis (n [%])	43 (59.72%)	45 (78.95%)	0.020
Interstitial inflammation (n [%])			<0.001
None	33 (45.83%)	0 (0.00%)	
Focal (<25%)	29 (40.28%)	46 (80.70%)	
Multifocal (25%< =∼<50%)	10 (13.89%)	11 (19.30%)	
Large patch (50%< =∼<75%)	0(0%)	0(0%)	
Diffuse (>70%)	0(0%)	0(0%)	
Fibrinoid necrosis (n [%])	7 (9.72%)	11 (19.30%)	0.119
Crescents (%, median (interquartile range))	0.00 (0.00-17.50)	6.67 (0.00-20.00)	0.412
Arteriole hypertrophy (n [%])	28 (38.89%)	38 (66.67%)	0.002

SLEDAI: systemic lupus erythematosus disease activity index; ds-DNA: double stranded DNA antibodies; C3, complement component 3; C4, complement component 4; eGFR: estimated glomerular filtration rate.

Histopathological analysis revealed that the tubular atrophy group had more pronounced global glomerulosclerosis (*p* < 0.001), segmental glomerulosclerosis (*p* = 0.02), mesangial hypercellularity (*p* = 0.041), endocapillary hypercellularity (*p* = 0.018), interstitial inflammation (*p* < 0.018), karyorrhexis (*p* = 0.02), and arteriole hypertrophy (*p* = 0.02).

### Univariable logistic regression model for tubular atrophy events

A univariable logistic regression analysis was conducted to identify potential risk factors for tubular atrophy events in patients with LN (Table 2). The analysis indicated that age [OR 1.057 (1.020, 1.094), *p* = 0.002], hypertension [OR 2.340 (1.125, 4.867), *p* = 0.022], leukocyte count [OR 1.123 (1.001, 1.260), *p* = 0.048], and serum C4 levels [OR 1.057 (1.012, 1.105), *p* = 0.013] were associated with an increased risk of tubular atrophy events in patients with LN. Conversely, higher hemoglobin levels [OR 0.983 (0.967, 0.999), *p* = 0.035] and higher eGFR [OR 0.986 (0.978, 0.995), *p* = 0.001] were associated with a reduced risk of tubular atrophy events.

**Table 2.** Potential Risk factors of tubular atrophy events in LN patients (univariable logistic regression analysis).

Exposure	OR	*p* Value
sex		
	Reference	
Sex, female	0.773 (0.235, 2.538)	0.67086
Age (years)	1.057 (1.020, 1.094)	0.00201
Systolic blood pressure (mmHg)	1.024 (1.005, 1.043)	0.01179
Diastolic blood pressure (mmHg)	1.027 (0.998, 1.056)	0.06778
Hypertension (yes/no)		
No	Reference	
yes	2.340 (1.125, 4.867)	0.02288
24h urinary protein (g)	1.108 (0.988, 1.243)	0.07997
Leukocyte (×10^9^ /L)	1.123 (1.001, 1.260)	0.04816
Hemoglobin (g)	0.983 (0.967, 0.999)	0.03589
Platelet (×10^9^ /L)	1.001 (0.997, 1.005)	0.64582
Serum albumin (g)	1.012 (0.969, 1.058)	0.57910
eGFR (mL/min/1.73 m^2^)	0.986 (0.978, 0.995)	0.00183
Serum uric acid (μmol/L)	1.002 (0.999, 1.004)	0.18938
LDL (mmol/L)	0.900 (0.708, 1.143)	0.38685
Serum C4 (0.01 g/L)	1.057 (1.012, 1.105)	0.01316
Serum C3 (g/L)	2.266 (0.625, 8.222)	0.21336
ANA		
0	Reference	
1:100	2.750 (0.162, 46.794)	0.48419
1:320	0.325 (0.027, 3.959)	0.37823
1:1000	0.364 (0.030, 4.366)	0.42501
1:3200	0.313 (0.025, 3.912)	0.36700
1:10000	0.227 (0.016, 3.131)	0.26824
SSA		
No	Reference	
Yes	1.548 (0.747, 3.208)	0.24033
SSB		
No	Reference	
Yes	1.123 (0.484, 2.602)	0.78718
Anti-ds-DNA		
No	Reference	
Yes	0.619 (0.282, 1.361)	0.23281
anti-SM antibody		
No	Reference	
Yes	0.568 (0.254, 1.273)	0.16956
SLEDAI score	1.015 (0.959, 1.074)	0.60552

ds-DNA: double stranded DNA antibodies; SLEDAI: systemic lupus erythematosus disease activity index; C3, complement component 3; C4, complement component 4; ANA: antinuclear antibodies; eGFR: estimated glomerular filtration rate.

### The relationship between C4 level and tubular atrophy events in patients with LN

A multivariable logistic regression model was conducted to investigate the association between serum C4 levels and tubular atrophy in patients with LN. After adjusting for age, sex, hypertension, 24-h urinary protein, hemoglobin, serum albumin, the proportion of global glomerulosclerosis, eGFR, the proportion of crescents, serum uric acid, the proportion of fibrinoid necrosis, endocapillary hypercellularity, anti-double stranded DNA antibodies, and anti-SM antibodies, serum C4 was identified as an independent risk factor for tubular atrophy in patients with LN (Table 3). Specifically, for every 0.01 g/L increase in serum C4, the risk of developing tubular atrophy events increased by 6.9%.

**Table 3.** The **r**elationship between C4 and tubular atrophy events in LN patients.

	Non-adjusted		Adjust I		Adjust II	
	OR	*p* Value	OR	*p* Value	OR	*p* Value
C4 per 0.01 g/L	1.057 (1.012, 1.105)	0.01316	1.069 (1.018, 1.122)	0.00754	1.069 (1.001, 1.141)	0.04817
C4 per 0.01 g/L tertile						
Low	Reference		Reference		Reference	
Middle	2.706 (1.105, 6.625)	0.02929	2.569 (0.977, 6.757)	0.05585	5.383 (1.434, 20.212)	0.01264
high	2.971 (1.212, 7.279)	0.01725	3.826 (1.439, 10.173)	0.00716	5.212 (1.232, 22.039)	0.02483
C4 per 0.01 g/L tertile for Linear trend test	1.701 (1.094, 2.643)	0.01826	1.941 (1.196, 3.152)	0.00731	2.236 (1.107, 4.516)	0.02489

Nonadjusted model adjust for: None.

Adjust I model adjust for: Age; Sex; Hypertension.

Adjust II model adjust for: Age; Sex; Hypertension;24h urinary protein; hemoglobin; serum albumin; the proportion of global glomerulosclerosis; eGFR; the proportion of crescents; Serum uric acid; the proportion of fibrinoid necrosis; endocapillary hypercellularity; anti-double stranded DNA antibodies; anti-SM antibody.

**Figure 1.**
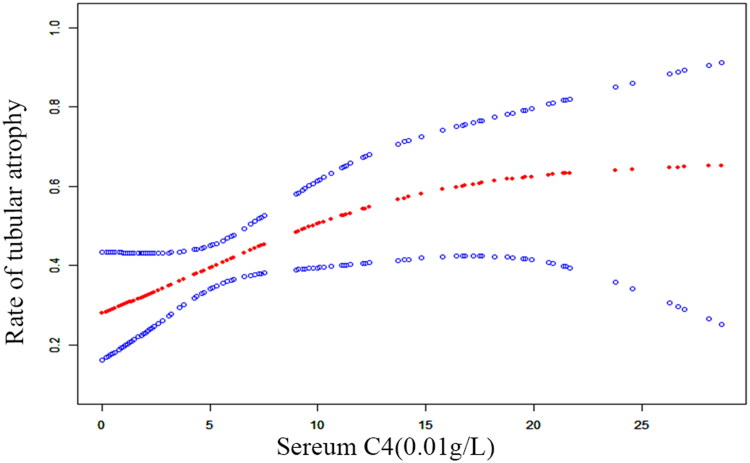
Spline smoothing plot of C4 and tubular atrophy.

A spline smoothing plot from a generalized additive mixed model analysis was also employed to examine the relationship between serum C4 levels and tubular atrophy in patients with LN. The plot demonstrated that an increase in serum C4 levels was associated with a higher risk of tubular atrophy in these patients (Figure 1).

### Stratified analysis between C4 level and tubular atrophy events in patients with LN

The relationship between serum C4 levels and tubular atrophy was further examined across various strata, including age, hypertension, leukocyte count, hemoglobin levels, and eGFR. The analysis revealed that this association remained consistent across these different stratifications (Table 4).

**Table 4.** The relationship between C4 level and tubular atrophy events under different stratifies.

	Number	OR (95%CI)	*P* value	*P* for interactions
Age (years)				0.1038
<30	59	1.027 (0.958, 1.101)	0.4594	
≥30	70	1.087 (1.013, 1.167)	0.0210	
hypertension				0.5705
no	82	1.050 (0.998, 1.105)	0.0592	
yes	47	1.129 (1.009, 1.263)	0.0346	
24h urinary protein (g/24 h)				0.7228
<3.5	87	1.074 (1.018, 1.133)	0.0093	
≥3.5	42	1.017 (0.931, 1.111)	0.7073	
Hemoglobin (g/L)				0.9079
<90	41	1.115 (0.981, 1.267)	0.0969	
> =90	88	1.062 (1.008, 1.118)	0.0229	
Serum albumin (g/L)				0.4399
<30	84	1.015 (0.947, 1.088)	0.6768	
≥30	45	1.096 (1.017, 1.181)	0.0167	
Leukocyte (×10^9^ /L)				0.9999
< =10	117	1.049 (1.002, 1.097)	0.0396	
>10	12	2.548 (0.787, 8.245)	0.1185	
eGFR (mL/min/1.73 m^2^)				0.0617
<90	59	1.149 (1.040, 1.270)	0.0064	
≥90	70	1.024 (0.975, 1.075)	0.3399	

adjust for: Age; Sex; Hypertension; 24h urinary protein; hemoglobin; Leukocyte; serum albumin; the proportion of global glomerulosclerosis; eGFR; the proportion of crescents; Serum uric acid; the proportion of fibrinoid necrosis; endocapillary hypercellularity; anti-double stranded DNA antibodies; anti-SM antibody.

## Discussion

In this study, analysis of clinical and histological features in 129 patients with LN revealed that tubular atrophy was present in 44% of the cases. This prevalence aligns with rates reported in previous studies [1,11]. The tubular atrophy group comprised older patients who were more likely to have hypertension. These patients also exhibited lower eGFR and hemoglobin levels, but higher serum C4 levels compared to the non-tubular atrophy group. Conversely, the non-tubular atrophy group had fewer instances of global and segmental glomerulosclerosis, mesangial hypercellularity, endocapillary hypercellularity, interstitial inflammation, karyorrhexis, and arteriole hypertrophy.

Previous studies have shown that in IgA nephropathy, lower plasma C4 levels are associated with reduced tubular atrophy as observed in renal biopsies [6]. However, there is limited research on the relationship between plasma C4 and tubular atrophy in patients with LN. In contrast, our study identified a significant correlation between serum C4 levels and the occurrence of tubular atrophy in patients with LN. Specifically, each 0.01 g/L increase in serum C4 was linked to a 6.9% higher risk of developing tubular atrophy. This result contrasts with previous studies, which reported no significant correlation between serum complement levels and interstitial fibrosis or tubular atrophy (IF/TA) [1,2,12,13]. The discrepancies between our findings and those of earlier studies may be attributed to variations in the classification of interstitial fibrosis and tubular atrophy or differences in sample sizes across the studies.

Extensive research has documented the complex roles of classical and alternative complement pathways in lupus nephritis pathogenesis. The immune complex-mediated activation of the classical complement pathway is crucial in driving tissue injury, which contributes to the development of renal tubular atrophy in lupus nephritis. Activation of the classical pathway is initiated by IgG or IgM bound to target antigens, and this pathway is activated in LN associated with the formation of immune complexes. In the classical pathway, lower C4 levels, but not C3 levels, significantly forecasted a renal flare. These observations suggest that the events that trigger renal flare in this immune complex disease involve classical pathway activation at least 2 months before clinical manifestation of the renal flare [14,15]. Paradoxically, this pathway also serves a protective function against autoimmunity; deficiencies in plasma C4 are recognized as independent risk factors for the development of SLE. The classical pathway facilitates the rapid clearance of apoptotic cells, thus preventing the immune system from mounting an autoimmune response against nuclear antigens. However, once autoimmunity is established, the activation of this pathway within the glomeruli can provoke downstream inflammatory responses [14]. This dual role of the pathway might explain the discrepancies between our study and others. Our research specifically focused on active LN, whereas previous studies often included both active and remissive phases of the disease. This variation in disease phase could contribute to the conflicting results regarding the association between serum complement C4 levels and tubular atrophy observed in renal biopsies [16].

In addition to the classical complement pathway, the alternative pathway may exacerbate LN by amplifying the activation of the classical pathway and its pro-inflammatory effects, leading to more severe renal damage. The alternative pathway is characterized by continuous low-level activation of plasma C3, resulting in the formation of C3b through a process known as ‘tickover’.And the finding that lower C3 levels significantly marked a renal flare in the absence of lower C4 levels suggests that the actual tissue damage that is manifested clinically as a renal flare involves amplified C3 activation [14], Since C4 is not involved in the alternative pathway, this could potentially obscure the relationship between C4 levels and tubular atrophy over time. Our study specifically focused on serum C4 and tubular atrophy at the onset of LN, whereas previous studies may have included cases at various stages of the disease, including both active and remissive phases. This difference in focus could account for the variation in results observed between our study and others.

The observed correlation between serum C4 levels and tubular atrophy in LN suggests a potential underlying mechanism that warrants further investigation. Additionally, given that IF/TA have been associated with poorer renal survival rates in patients with LN according to previous studies, it is hypothesized that elevated serum C4 levels at the time of biopsy might be linked to reduced eGFR values due to an increased risk of tubular atrophy [16]. This hypothesis requires validation in future research to confirm the relationship.

As one of the most prevalent forms of secondary nephritis, LN is a significant contributor to the progression toward ESRD. Tubular atrophy has been identified as an independent risk factor for poor renal outcomes in patients with LN [1,2]. However, there is limited understanding of the clinical features associated with tubular atrophy in patients with LN.

Complement, an essential component of the innate immune system, is tightly regulated by various fluid-phase and cell surface proteins to prevent damage to autologous tissues. It plays a critical role in defending against microbial infections and clearing immune complexes and damaged cells. In LN, intrinsic kidney antigens bind to autoantibodies as well as deposited antigens, potentially triggering complement activation. The classical pathway of complement activation, initiated by deposited immune complexes, may lead to a cascade of intra-renal complement activation. Hyperactivation of complement in LN drives severe inflammatory responses in the kidneys [17]. The membrane attack complex, the terminal product of complement activation, forms pores in plasma membranes, allowing ions to pass through and causing activation or lysis of target cells [14]. Previous research has shown that the complement system is involved in multiple pathogenic processes in SLE [18]. Due to a relative deficiency in complement regulators, renal tubules are particularly susceptible to complement-mediated damage, which may contribute to tubular atrophy [17].

In addition, the study has some limitations. First, the lack of follow-up data prevents us from understanding the long-term implications of the observed relationship between C4 levels and tubular atrophy. Second, treatment information was not included in the analysis, which may be an important confounding factor, as different treatment regimens may affect the development of tubular atrophy. Future prospective studies should address these limitations by including long-term follow-up data and detailed treatment information. In addition, this study is a single-center, retrospective cross-sectional study, which has potential bias that may affect the generality of the findings. The study population was composed primarily of Asian patients, which can lead to demographic bias. Therefore, a well-designed prospective study is needed to validate these results.

In conclusion, tubular atrophy in LN may result from multiple contributing factors. This study demonstrates a positive association between serum C4 levels at the time of biopsy and the risk of tubular atrophy in patients with LN. Further research is needed to elucidate the underlying mechanisms that link serum C4 levels to the development of tubular atrophy in LN.

## Acknowledgements

We would like to acknowledge the hard and dedicated work of all the staff that implemented the intervention and evaluation components of the study.

## Supplementary Material

Supplementary_table_1.docx

## Data Availability

All data generated or analyzed during this study are included in this article. Further enquiries can be directed to the corresponding author.
