# Supplementary material for: Correlation between serum complement component 4 levels and tubular atrophy in patients with lupus nephritis
Source: Ren Fail. 2025 Mar 17;47(1):2477833. doi: 10.1080/0886022X.2025.2477833 (PMC11915727; doi:10.1080/0886022X.2025.2477833)
Supplement: Supplementary_table_1.docx [file IRNF_A_2477833_SM0199.docx]

Table S1 variance inflation factor (VIF) analysis of the variable

|  | Perform variance inflation factor (VIF) |
| --- | --- |
| sex | 1.2 |
| Age | 1.6 |
| Hypertension | 1.3 |
| hemoglobin | 1.6 |
| serum albumin | 1.4 |
| the proportion of global glomerulosclerosis | 1.6 |
| eGFR | 4 |
| the proportion of crescents | 2.1 |
| Serum uric acid | 2.3 |
| the proportion of fibrinoid necrosis | 1.3 |
| endocapillary hypercellularity | 1.6 |
| anti-double stranded DNA | 1.4 |
| anti-SM antibody | 1.2 |
| CRP | 1.2 |
